# Supplementary material for: Robotic modelling of snake traversing large, smooth obstacles reveals stability benefits of body compliance
Source: R Soc Open Sci. 2020 Feb 19;7(2):191192. doi: 10.1098/rsos.191192 (PMC7062058; doi:10.1098/rsos.191192)
Supplement: Supplementary Material text file (containing Materials and Methods, Fig. S1-S3, and Table S1). [file rsos191192supp1.pdf]

Robotic modeling of snake traversing large, smooth obstacles reveals stability benefits of body compliance

Supplemental Material

Royal Society Open Science

Qiyuan Fu, Chen Li*

Department of Mechanical Engineering, Johns Hopkins University, Baltimore, Maryland, USA

*Corresponding author. E-mail: chen.li@jhu.edu

Materials and Methods

Robot parts

The robot was actuated with 19 Dynamixel XM430-W350-R servo motors operating at 14 V, powered by an external DC power supply (TekPower, CA, USA). The rubber O-rings wrapping each wheel were oil-resistant soft buna-n O-rings with an outer diameter of 48.1 mm and a width of 5.3 mm (McMaster-Carr, Elmhurst, IL, USA). The springs used in the suspension were compression springs with a length of 9.5 mm and an outer diameter of 3.1 mm (McMaster-Carr, Elmhurst, IL, USA). The maximal compression of each spring was 5 mm, which, when amplified by the lever arm (Fig. 2, red), limited the suspension deformation of each wheel to within 10 mm.

Large step obstacle track

We constructed a 180 cm long, 120 cm wide obstacle track using extruded T-slotted aluminum and acrylic sheets (McMaster-Carr, Elmhurst, IL, USA) (Fig. S1A). The step spanned the entire width of the track. To reduce slipping of the robot, we covered the horizontal surfaces of the step with a high friction rubber sheet (EPDM 60A 1.6 mm thick rubber sheet, Rubber-Cal, CA, USA).

Friction measurement

In friction experiments, we measured the position as a function of time of three body segments being dragged by a weight, by tracking ArUco tags in videos captured by Logitech C920 webcam at 30 frames/s. Then, by fitting a quadratic function of displacement as a function of time to estimate acceleration, we calculated kinetic friction coefficient as:

$$\mu = \frac{m_2 g - (m_1 + m_2)a}{m_1 g}$$

where m_1 is the mass of the weight, m_2 is the total mass of the segments, a is the fitted acceleration, g is the local gravitational acceleration (9.81514 m/s^2).

Motor actuation to achieve partitioned gait

The actuation profile of yaw joints in the laterally undulating body sections, defined as the angular displacement from the straight body pose (Fig. 2B, yellow angle) as a function of time and segment index, followed the serpenoid gait [18]:

$$\theta_i = \begin{cases} A sin(\omega t + \phi + (i-1)\Delta\phi), i = 1,2, \dots, k_1 \\ A sin(\omega t + \phi + (i + k_1 - k_2)\Delta\phi), i = k_2, \dots, 9 \end{cases}$$

where i is for the ith yaw joint from the robot head, $A = \pi/6$ and $\omega = \pi/2$ are the amplitude and angular velocity of each yaw joint angle waveform, $\phi = 0$ is the initial phase (at time zero) of the first yaw joint, and $\Delta\phi = -\pi/4$ is the phase difference between adjacent yaw joints. $\Delta\phi$ determines the wavenumber of the entire serpenoid wave in the robot, $k = 9|\Delta\phi|/2\pi$. The k_1th yaw joint is the last yaw joint in the undulating section above the step, and the k_2th yaw joint is the first yaw joint in the undulating section below the step, $k_2 - k_1$ is the number of pitch segments in the cantilevering section. The pitch angles of all pitch segments in these two undulating sections were set to zero (Fig. S2A, gray) to maintain contact with the horizontal surfaces.

The actuation profile of the joints of the cantilevering section (Fig. S2A, red) was designed to bridge across the large step with the minimal number of segments necessary. The yaw angles of all yaw segments in this section were set to zero. The pitch angle of the most anterior pitch joint in the undulating section below (Fig. S2A, joint c) was set to its maximal possible value ϕ_{max} so that the cantilevering section was as vertical as possible to minimize cantilevering length. The two most anterior pitch joints in the cantilevering section (Fig. S2A, joints a and b) were set to keep the section above in contact with the upper horizontal surface. Their pitch angles were calculated as follows: $\phi_a = \phi_b - \phi_{max}$, $\phi_b = -\sin^{-1}[(H - nh\sin\phi_{max})/L]$, $n = \text{floor}[H/(h\sin\phi_{max})]$, where H is step height, h is the distance between two adjacent pitch

axes when the robot is straight, n the maximum number of pitch and yaw segments that can be kept straight in the cantilevering section.

Marker-based feedback logic control

For feedback logic control [40] of the robot, a 3 × 3 cm ArUco marker [64] was fixed to the top of each pitch segment and on both the upper and lower horizontal surfaces near the top and bottom edge of the step (Fig. S1B). Their positions captured by a camera were tracked before each trial to measure the step height for adjusting the robot gait and then tracked online to locate the position of each pitch segment relative to the step. We used a webcam (C920, Logitech, Lausanne, Switzerland) with 1920 × 1080 resolution for experiments with step height $H \leq 38\% \, L$. We used another camera (Flea3, FLIR, OR, USA) with 1280 × 1024 resolution and a 12.5 mm lens (Fujinon CF12.5HA-1, Fujifilm, Minato, Japan) for experiments with $H > 38\% \, L$ because the webcam could not capture the entire setup with its limited focus length and angle of view.

The snake robot was controlled by a custom Robot Operating System (ROS) package running on an Ubuntu laptop connected with the online camera and a power sensor system to measure electrical power consumption (see below) (Fig. S2B). The laptop sent joint position commands to the servo motors and received motor angle readings at around 20 Hz. The online camera sent images to the laptop for online tracking of the ArUco markers at 20 Hz.

The feedback logic control algorithm is shown in flow chart (Fig. S2C). Before entering the main loop of online servo motor control at 25 Hz (in ROS time), the actuation profile of pitch segments was first calculated based on the step height acquired. In each control loop, the controller determined whether section division needed to be propagated down the body by checking: (1) whether the middle point of the motor axle line segment of the most posterior pitch segment in the cantilevering section had crossed a vertical plane 4 cm before and parallel to the vertical surface of the step but was no higher than 10 cm above the upper horizontal surface; or (2) whether the middle point of the motor axle line segment of the most anterior pitch segment in the undulating section below the step had crossed a vertical plane 12 cm before and parallel to the vertical surface of the step. If either was true, the controller calculated the updated joint angles and

sent angle commands to the servo motors. The controller continued this loop until a termination signal sent by the experimenter was received.

Electrical power measurement

We used two current sensors (Adafruit, NY, USA) between the servo motors and the power supply to record both voltage and current and measure electrical power of the robot (Fig. S3) at 100-135 Hz. The two sensors were installed on the power cord near the power supply in parallel to accommodate the large current drawn. The DC current and voltage data were sent to the laptop for recording with timestamps via an Arduino-based Single Chip Processor (SCP) communicating with the laptop.

Data synchronization

To synchronize motor angle data and electrical power data recorded by the laptop with the high-speed camera videos recorded on a desktop server, the power measurement circuit included a switch to turn on/off an LED bulb placed in the field of view of the high-speed cameras. When the LED was switched on/off, the SCP detected the voltage increase/drop and began/stopped recording power data. By aligning the initial and final power data points with the LED on/off frames in the videos and interpolating the motor position and electrical power data to the same sampling frequency as high-speed video frame rate (100 Hz), these data were synchronized.

Experiment protocol

At the beginning of each trial, we placed the robot on the surface below the step at the same initial position and orientation. The robot was set straight with its body longitudinal axis perpendicular to the vertical surface of the step. Its distance was set to be 16.5 cm from the wheel axle of the first segment to the vertical surface. This distance was selected so that the forward direction of most anterior segment in the undulating section below the step was perpendicular to the vertical surface before it began to cantilever. We then started high-speed video recording and switched on the LED in the SCP circuit. Next, we started the robot motion and monitored traversal progress until a termination condition was met. After the robot motion was terminated, the LED was first switched off, then the high-speed camera recording was stopped, and the setup was reset for the next trial while high speed videos were saved.

3-D kinematics reconstruction

To reconstruct 3-D kinematics of the entire robot traversing the large step obstacle, we recorded the experiments using twelve high-speed cameras (Adimec, Eindhoven, Netherlands) with a resolution of 2592 × 2048 pixels at 100 frames s^{-1} (Fig. S1A). The experiment arena was illuminated by four 500 W halogen lamps and four LED lights placed from the top and side.

To calibrate the cameras over the entire working space for 3-D reconstruction, we built a three section, step-like calibration object using T-slotted aluminum and Lego Duplo bricks (The Lego Group, Denmark). The calibration object consisted of 23 landmarks with 83 BEEtags [65] facing different directions for automatic tracking. We then used the tracked 2-D coordinates of the BEEtag center points for 3-D calibration using Direct Linear Transformation (DLT) [66]. To obtain 3-D kinematics of the robot relative to the step, we used the 10 ArUco markers attached to the robot (one on each pitch segment), the two attached near the top and bottom edge of the step, and 13 additional ones temporarily placed on the three step surfaces before the first trial of each step height treatment. After all the experiments, we used a custom C++ script to track the 2-D coordinates of the corner points of each ArUco marker in each camera view. We checked and rejected ArUco tracking data whose four marker corners did not form a square shape with a small tolerance (10% side length).

Using the tracked 2-D coordinates from multiple camera views, we obtained 3-D coordinates of each tracked marker via DLT using a custom MATLAB script. We rejected marker data where there was an unrealistic large acceleration (> 10 m/s^2), resulting from a marker suddenly disappearing in one camera view while appearing in another in the same frame. We then obtained 3-D position and orientation of each pitch segment by offsetting its marker 3-D position and orientation using the 3-D transformation matrices from the marker to the segment, which was measured from the CAD model of the robot. We also measured the step geometry by fitting a plane to the markers on each of its three surfaces and generated a point cloud using the fit equation and the dimension of the three surfaces.

For yaw segments without markers and the pitch segments whose markers were not tracked due to occlusions or large rotation, we inferred their 3-D positions and orientations using kinematic constraints.

We first tried inputting motor angles recorded by these segment motors into the robot forward kinematics to solve for their transformation matrices from other reconstructed segments. If their motor angles were not properly recorded, we tried inferring their positions and orientations from the two adjacent segments (as long as they were reconstructed). To do so, we first obtained all servo motor angles in this missing section by solving an inverse kinematics problem, then derived the transformation matrices of the missing segments from the forward kinematics. Finally, if both methods failed, we interpolated temporally from adjacent frames to fill in the missing transformation matrices. The interpolation was linearly applied on the twists of transformation matrices. We compared joint angles from the reconstructed segments to motor position data and rejected those with an error larger than 10°. To reduce high frequency tracking noise, we applied a window average filter temporally (*smooth2a*, averaging over 11 frames) to the 3-D positions of each segment after reconstructing all segments.

We verified the fidelity of 3-D kinematics reconstruction by projecting reconstruction back onto the high-speed videos and visually examined the match (Fig. S1B). The thresholds used in this process were selected by trial and error, with the aim of removing substantial visible projection errors while rejecting as few data as possible.

Data analysis

To quantify traversal performance, we measured traversal probability defined as the ratio of the number of trials in which the entire robot reached the surface above the step to the total number of trials for each step height. To quantify roll instability, we measured roll failure (flipping over) probability, defined as the ratio of the number of failed trials in which the robot flipped over due to rolling to the total number of trials for each step height. To determine whether a wheel contacted a surface, we examined whether any point in the wheel point cloud (Fig. 8B, grey dashed circle) penetrated the surface assuming no suspension compression. Unrealistic body deformation values from tracking errors larger than the 10 mm limit from the mechanical structure were set to 10 mm.

To compare electrical power during traversal across step height and body compliance treatments, we analyzed electrical power over the traversal process, defined as from when the first pitch segment lifted

to cantilever, to when the last pitch segment crossed the top edge of the step for successful trials, or to when the robot flipped over (roll failure) or the trial was terminated due to robot getting stuck (stuck failure) for failed trials.

To compare traversal performance of our robot with previous snake robots and the kingsnake, we calculated vertical traversal speed for each robot and the animal. For our snake robot and the kingsnake with multiple trials, we first calculated vertical traversal speed of each trial by dividing step height normalized to body length by traversal time and then pooled speed data of all trials from all step heights for each body compliance treatments (for the robot) to obtain average speed. The slopes shown in Fig. 9 are average vertical traversal speed for each robot and the animal.

During experiments, we rejected trials in which the robot moved out of the obstacle track before successfully traversing the step or failing to traverse due to occasional crash of the control program. We collected around 10 trials for each combination of step height and suspension setting (rigid and compliant). For the rigid body, 40% *L* step treatment only 5 trials were collected, because the 3-D printed segment connectors were often damaged by ground collisions during roll failure (flipping over) and had to be replaced. Detailed sample size is shown in Table S1.

Table S1. Sample size.

	H = 31% *L*	*H* = 36% *L*	*H* = 38% *L*	*H* = 40% *L*
Rigid	10	8	10	5
Compliant	10	11	10	10

Records of traversal success and roll failure (flipping over) were binomial values (1 for success and 0 for failure) for each trial and averaged across trials to obtain their probabilities for each step height and body compliance treatment. For each trial, contact probability, body deformation, and surface conformation difference were averaged spatiotemporally over time and across all pitch segments in the undulating sections above and below the step combined. Electrical power was averaged over time for each trial. Finally, these trial averages were further averaged across trials for each step height and body compliance treatment

to obtain treatment means and standard deviations (s.d.) or confidence intervals, which are reported in figures.

To test whether traversal probability and roll failure (flipping over) probability depended on step height, for the rigid or compliant body robot, we used a simple logistic regression separately for each of these measurements, with step height as a continuous independent factor and records of traversal success or roll failure (flipping over) as a nominal dependent factor.

To test whether traversal probability and roll failure (flipping over) probability further depended on body compliance while taking into account the effect of step height, we used a multiple logistic regression for each of these measurements with data from rigid and compliant body robot combined, with body compliance as a nominal independent factor and step height as a continuous independent factor and records of traversal success or roll failure (flipping over) as a nominal dependent factor.

To test whether traversal probability differed between each adjacent pair of step heights for the rigid or compliant body robot, we used a pairwise chi-square test for each pair of step heights, with step height as a nominal independent factor and traversal success record as a nominal dependent factor.

To test whether contact probability, body deformation, surface conformation difference, and electrical power differed between rigid and compliant body robot, we used an ANCOVA for each of these measurements. We first set body compliance, step height, and their interaction term as independent factors and each of these measurements as a nominal/continuous dependent factor. If the P value of the interaction term was less than 0.05, we then re-ran the same test excluding the interaction term.

All the statistical tests followed the SAS examples in [67] and were performed using JMP Pro 13 (SAS Institute, Cary, NC, USA).

Supplementary Figures

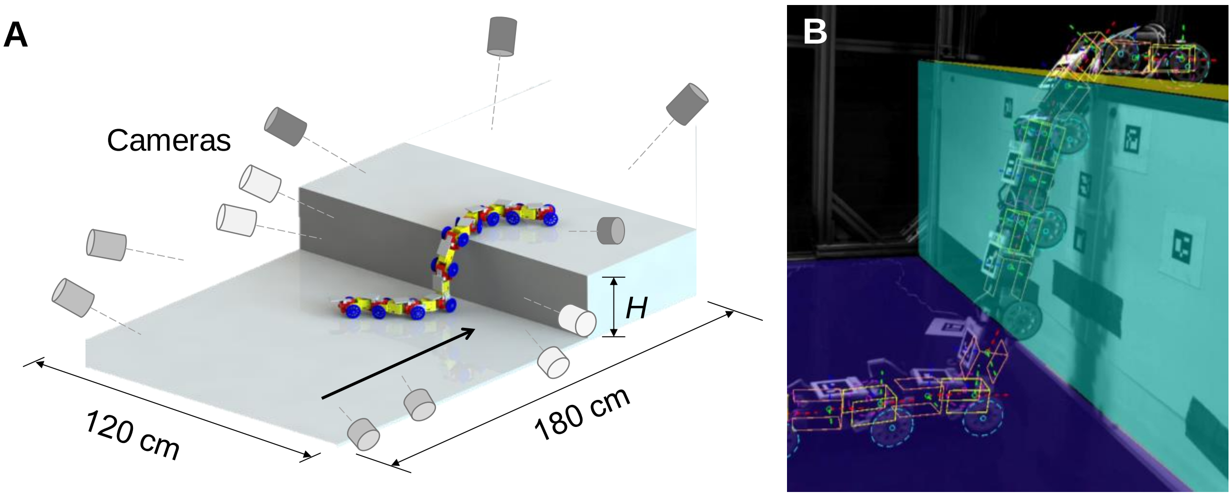

Fig. S1. Experimental setup and 3-D kinematics reconstruction. (A) Schematic of experimental setup. Twelve high-speed cameras are used for 3-D kinematics reconstruction, divided into groups of four (different shades) focusing on three step surfaces. **(B)** High-speed video snapshot of robot traversing step, with projection of reconstructed body segments, wheels, and step surfaces. Yellow and orange boxes are reconstructed yaw and pitch servo motors. Dashed magenta and cyan circles are reconstructed left and right wheels assuming no suspension compression. Violet, cyan, and gold surfaces are reconstructed lower horizontal, vertical, and upper horizontal surfaces.

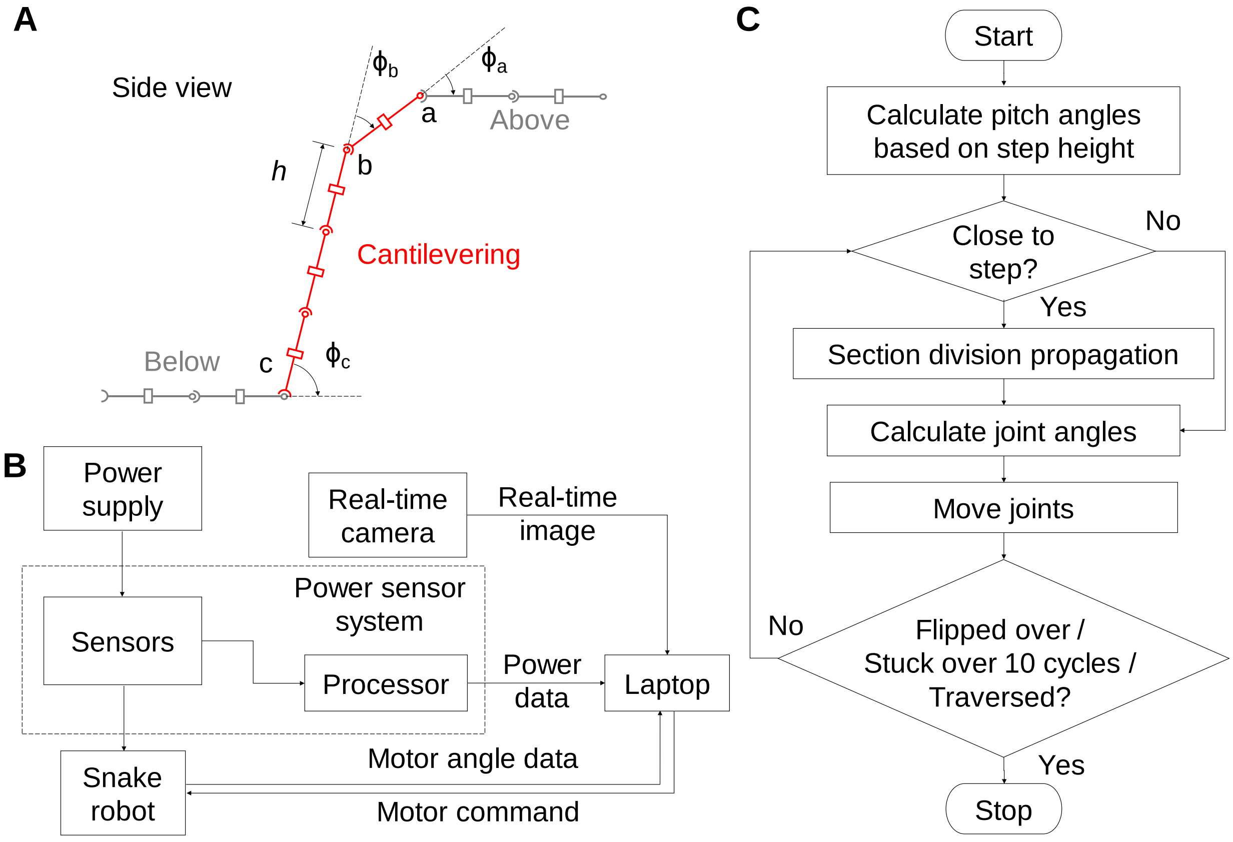

Fig. S2. Controller design. (A) Side view schematic of partitioned gait design to show control of cantilevering section (red). Three pitch angles are calculated based on measured step height, including: ϕ_a and ϕ_b of the two most anterior pitch joint of the cantilevering section and ϕ_c of the most anterior pitch joint of the undulating section below the step. **(B)** Data acquisition system. **(C)** Flow chart of robot control. For (B) and (C), see **Section 2.2** in main text and **Marker-based feedback logic control** and **Electrical power measurement** in Materials and Methods for detailed description.

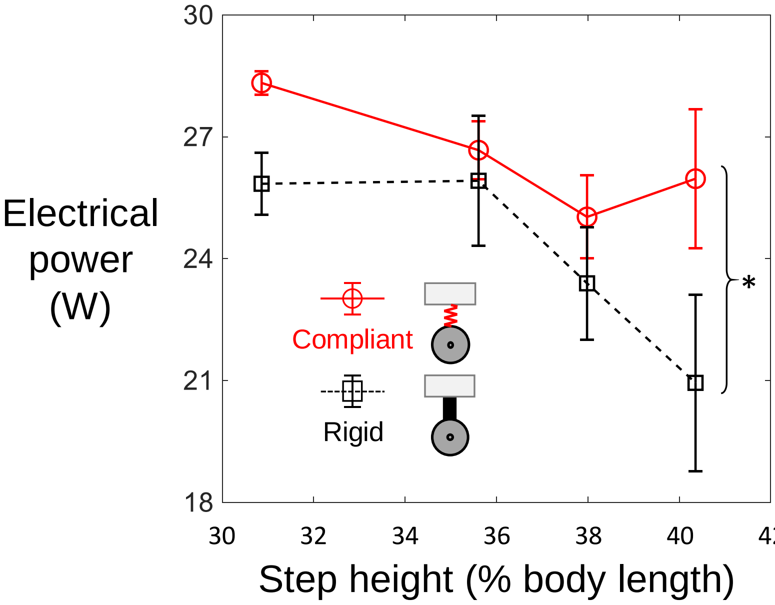

Fig. S3. Effect of body compliance on electrical power. Electrical power of robot as a function of step height. Black dashed is for rigid body robot; red solid is for compliant body robot. Error bars show ± 1 s.d. Bracket and asterisk represent a significant difference between rigid and compliant body robot ($P < 0.0001$, ANCOVA).

Supplementary Movies

Movie 1. Mechanical design of snake robot.

Move 2. Comparison of large step traversal between rigid and compliant body snake robot.

Movie 3. Adverse events of snake robot traversing a large step.
